# Inhibition of HIV-1 Replication in Human Monocyte-Derived Macrophages by Parasite *Trypanosoma cruzi*

**DOI:** 10.1371/journal.pone.0008246

**Published:** 2009-12-14

**Authors:** Guadalupe Andreani, Ana M. Celentano, María E. Solana, Silvia I. Cazorla, Emilio L. Malchiodi, Liliana A. Martínez Peralta, Guillermina L. Dolcini

**Affiliations:** 1 National Reference Center for AIDS, Microbiology Department, School of Medicine, University of Buenos Aires, Buenos Aires, Argentina; 2 Laboratory of Parasitology, Microbiology Department, School of Medicine, University of Buenos Aires, Buenos Aires, Argentina; 3 IDEHU–Institute of Studies on Humoral Immunity, CONICET-UBA, School of Pharmacy and Biochemistry, University of Buenos Aires, Buenos Aires, Argentina; Federal University of São Paulo, Brazil

## Abstract

**Background:**

Cells of monocyte/macrophage lineage are one of the major targets of HIV-1 infection and serve as reservoirs for viral persistence *in vivo*. These cells are also the target of the protozoa *Trypanosoma cruzi*, the causative agent of Chagas disease, being one of the most important endemic protozoonoses in Latin America. It has been demonstrated *in vitro* that co-infection with other pathogens can modulate HIV replication. However, no studies at cellular level have suggested an interaction between *T. cruzi* and HIV-1 to date.

**Methodology/Principal Findings:**

By using a fully replicative wild-type virus, our study showed that *T. cruzi* inhibits HIV-1 antigen production by nearly 100% (*p<*0.001) in monocyte-derived macrophages (MDM). In different infection schemes with luciferase-reporter VSV-G or BaL pseudotyped HIV-1 and trypomastigotes, *T. cruzi* induced a significant reduction of luciferase level for both pseudotypes in all the infection schemes (*p<*0.001), *T. cruzi*-HIV (>99%) being stronger than HIV-*T. cruzi* (∼90% for BaL and ∼85% for VSV-G) infection. In MDM with established HIV-1 infection, *T. cruzi* significantly inhibited luciferate activity (*p*<0.01). By quantifying R-U5 and U5-gag transcripts by real time PCR, our study showed the expression of both transcripts significantly diminished in the presence of trypomastigotes (*p<*0.05). Thus, *T. cruzi* inhibits viral post-integration steps, early post-entry steps and entry into MDM. Trypomastigotes also caused a ∼60-70% decrease of surface CCR5 expression on MDM. Multiplication of *T. cruzi* inside the MDM does not seem to be required for inhibiting HIV-1 replication since soluble factors secreted by trypomastigotes have shown similar effects. Moreover, the major parasite antigen cruzipain, which is secreted by the trypomastigote form, was able to inhibit viral production in MDM over 90% (*p<*0.01).

**Conclusions/Significance:**

Our study showed that *T. cruzi* inhibits HIV-1 replication at several replication stages in macrophages, a major cell target for both pathogens.

## Introduction

Both HIV-1 infection course and outcome are determined by the interaction between viral and host cellular factors [1], as well as by additional agents - termed cofactors - that may have an influence on the progression and transmission of the infection. Since numerous tropical pathogens lead to opportunistic infections in the context of HIV, co-infection could have significant effects on the course of HIV infection [2], [3]. Some of these pathogens are able to infect the same cells as HIV; thus, they may be considered as putative cofactors in the course of HIV infection. Cells of monocyte/macrophage lineage are among the first cells to be infected with HIV-1 and may also persist in tissues for long periods of time and contribute to the spread of viral infection [4]–[6]. It has been demonstrated *in vitro* that macrophage co-infections with *Mycobacterium tuberculosis* [7] or *Leishmania infantum* [8], [9] may modulate the expression of certain factors that are able to modify HIV-1 replication.

The parasite *Trypanosoma cruzi* (*T. cruzi*) causes Chagas disease, one of the most important endemic protozoonoses in Latin America. Chagas disease is characterized by an acute phase, with high, generally self-limited parasitemia followed by an undetermined phase that can last for years without signs or symptoms. After this phase, between 20 and 30% of the patients advance to a chronic phase, where different events of cardiopathy or megaviscera may occur. There are approximately 16–18 million infected individuals, representing the largest burden of vector-borne parasitic diseases in the continent, with around 50,000 deaths per year and 100 million subjects at risk of infection [10]–[12]. Largely considered as a rural entity, Chagas disease has become an urban public health concern due to the massive migration of rural populations to big cities, including cities in America and Europe [13]. These changes in the epidemiology of Chagas disease facilitate co-infection with HIV-1 in areas with high viral prevalence. In endemic and epidemiologic areas, *T. cruzi* infection occurs at an early age and HIV co-infection mostly happens when the patient goes through the undetermined or chronic phase, where the parasite is confined mostly to non-phagocytic cells [14], [15]. Data from *T. cruzi*-HIV co-infected patients indicate reactivation of parasite infection with exacerbation of clinical signs and unusual clinical manifestations when the patient is undergoing immunosuppression [16]–[18].

Cell infection with *T. cruzi* begins with the uptake of infective trypomastigotes within phagosomes and their release into the cytosol, where they are transformed into replicating amastigotes; the latter, in turn, differentiate into trypomastigotes and are released during cytolysis. The parasite invades and multiplies inside inactivated macrophages and dendritic cells in early infection and can be carried by these cells to targeted tissues. Activated macrophages are involved in early parasite killing [10]. *T. cruzi* expresses and/or secretes effector molecules that control cell entry and intracellular targeting or modulate host cell functions required for survival, multiplication, and dissemination in the host [19]–[22]. Among them, cruzipain - the major lysosomal cysteine proteinase [23], [24] - proved to play a crucial role in Chagas disease, including the process of parasite internalization within mammalian cells [25].

Recently, we described the inhibition of HIV replication by *T. cruzi* in a human placental model [26]. However, no *in vitro* studies have described the interaction between *T. cruzi* and HIV-1 in a relevant cell type for both pathogens to now. Since both of them are able to infect and replicate within macrophages, we analyzed whether *T. cruzi* affects the HIV-1 replication cycle in human monocyte-derived macrophages. Our study shows that *T. cruzi* trypomastigotes and the soluble factors shed by them impair HIV-1 replication in MDM at different stages.

## Results

### *T. cruzi* Inhibits HIV-1 Replication in Different Cell Types

**Figure 1:**
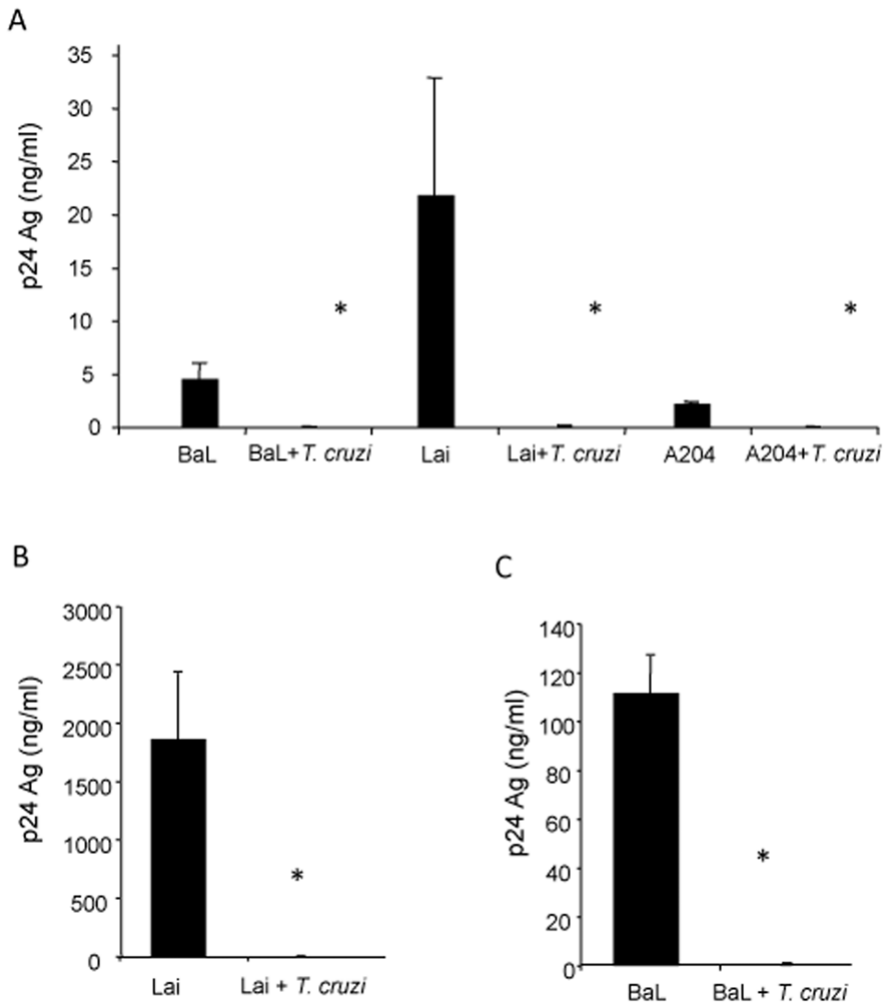
Inhibition of HIV-1 replication by *T. cruzi* in different cell types. Cells were infected with HIV-1 primary isolates with or without *T. cruzi* blood trypomastigotes and p24 production was measured at day 8 p.i. (**A**) PMBCs infected with R5 (BaL), X4 (Lai) and R5X4 (A204). (**B**) SupT-1 cell line infected with X4 (Lai) isolate and (**C**) MDM infected with R5 (BaL). Results are expressed as mean ± SD, and are representative of at least 2 independent experiments. * *p*<0.001.

The effect of *T. cruzi* blood trypomastigotes on HIV replication was evaluated for PBMCs, MDM and the T-lymphoblastoid cell line SupT-1 using R5 (BaL), X4 (HXB2) and dual tropic (A204) isolates. Preliminary experiments showed that a parasite/cell ratio of 5∶1 or greater equally inhibited p24 antigen production, while a lower number of parasites per cell inhibited less than 100%. Accordingly, the 5∶1 parasite/cell ratio was chosen for all the experiments in this study. Cells were infected overnight with a parasite/cell ratio of 5∶1 and virus at the same time. P24 antigen production was evaluated in culture supernatant 8 days p.i. (**Fig. 1**). Viral production was significantly reduced in the three cell types used (*p*<0.001). These results show that *T. cruzi* inhibits HIV-1 replication in different cell types. Since macrophages are among the most relevant cells involved in HIV-1 and *T. cruzi* infection, the analysis of the steps affected by co-infection was conducted on MDM.

### *T. cruzi* Trypomastigotes and Parasitic Soluble Factors Inhibit HIV-1 Production in MDM

**Figure 2:**
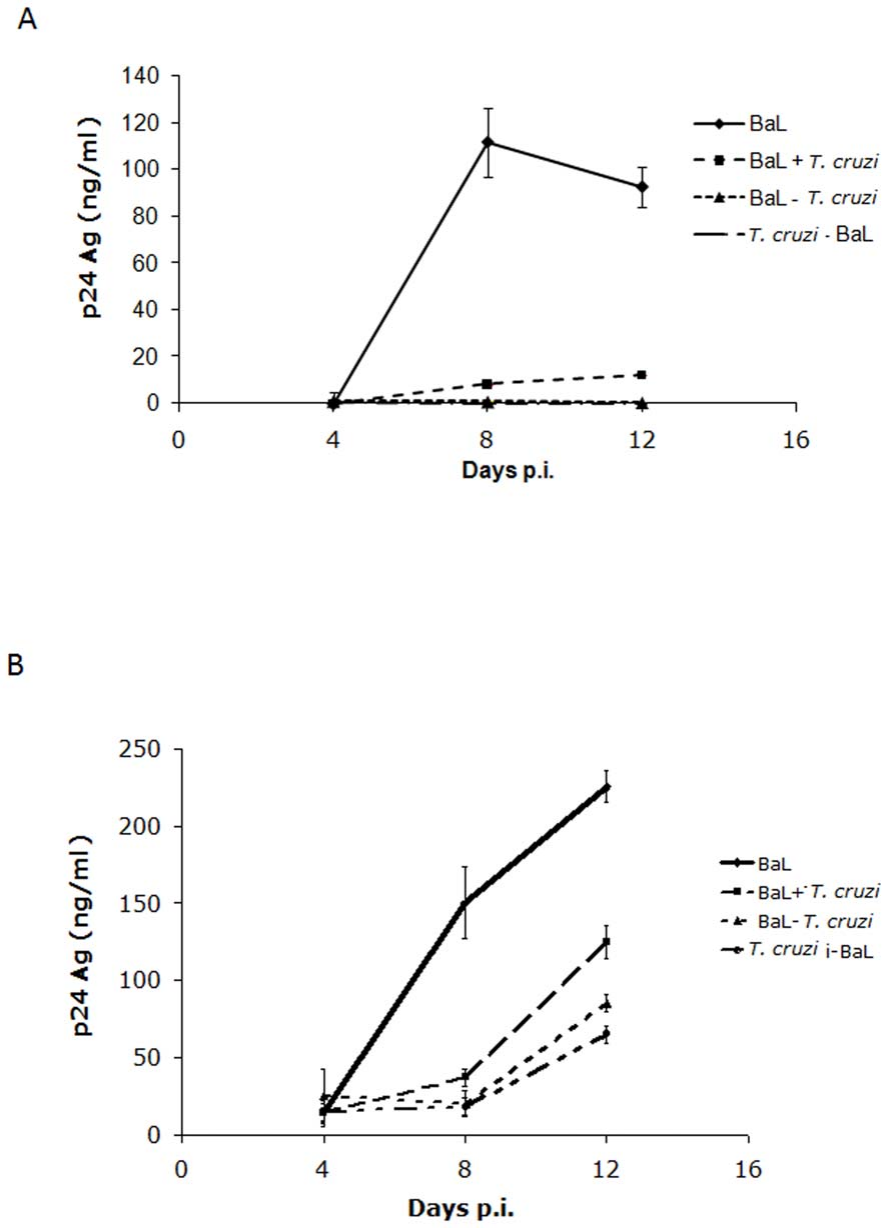
Inhibition of HIV-1 production by *T. cruzi* in MDM. MDM were infected with HIV in presence of *T. cruzi* blood trypomastigotes (**A**) or trypomastigotes free-supernatant (TcSn) (**B**) in three different schemes: HIV 24 h after *T. cruzi* (*T. cruzi*-HIV), HIV 24 h before *T. cruzi* (HIV-*T. cruzi)* or HIV at the same time as *T. cruzi* (HIV+*T. cruzi*), where *T. cruzi* indicates either trypomastigotes or TcSn. P24 antigen production was measured at days 4, 8 and 12 p.i. in culture supernatants. Results are expressed as mean ± SD of triplicates of infection and a representative experiment of at least 3 independent experiments performed with cells from different donors is shown.

To begin to elucidate which steps of the HIV-1 replication cycle were affected by the parasite, MDM were infected with HIV-1_BaL_ and blood trypomastigotes using three different schemes: HIV 24 h before *T. cruzi* (HIV-*T. cruzi)*, HIV 24 h after *T. cruzi* (*T. cruzi*-HIV), or HIV at the same time as *T. cruzi* (HIV+*T. cruzi*). P24 antigen production in culture supernatants was measured at days 4, 8 and 12 post-viral infection. The results indicate that HIV replication is inhibited by *T. cruzi* trypomastigotes nearly by 100%, regardless of the infection scheme (*p<*0.001) (**Fig. 2A**). To determine whether active infection of MDM with *T. cruzi* was necessary for viral replication impairment, co-infections were carried out in the same schemes as described above using excreted/secreted antigens (TcSn), obtained as described in Material and Methods. Results showed that TcSn were also able to inhibit p24 production (*p<*0.001), regardless of the scheme used (**Fig. 2B**). However the inhibition tended to be lost with time, as the p24 production began to increase at day 8, whereas in the presence of live trypomastigotes no production of p24 was detected throughout this study (**Fig. 2A**). This could be attributed to the fact that cells were treated with the TcSn at the beginning of the experiment, while the trypomastigotes were present throughout the experiment, as they replicate in the cultures.

### *T. cruzi* Inhibits Pseudotyped Virus Replication

**Figure 3:**
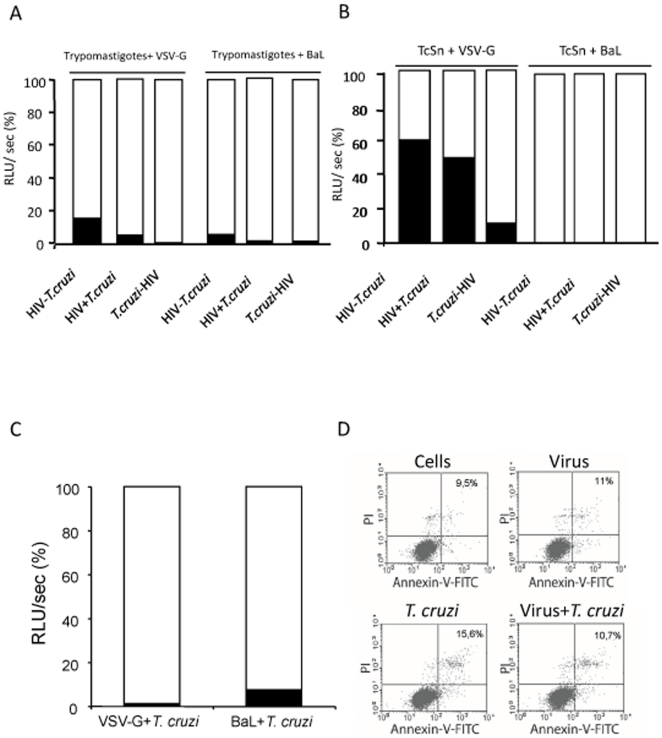
Inhibition of pseudotyped viruses replication by *T. cruzi.* MDM were infected with both VSV-G or BaL pseudotyped viruses in the presence of *T. cruzi* blood trypomastigotes (**A**) or trypomastigotes free-supernatant (TcSn) (**B**) in three different schemes: HIV 24 h after *T. cruzi* (*T. cruzi*-HIV), HIV 24 h before *T. cruzi* (HIV-*T. cruzi*) or HIV at the same time as *T. cruzi* (HIV+*T. cruzi*), where *T. cruzi* indicates either trypomastigotes of TcSn. Luciferase activity was measured from cell lysates 4 days post-viral infection. Results are expressed as relative light units per second (RLU/sec), presented as a percentage relative to the control (100%), where the histogram in white corresponds to the percentage of infection with the respective control virus and the histogram in black corresponds to the percentage of infection in the presence of the parasite. (**C**) MDM were also infected with pseudotyped viruses and cell-derived trypomastigotes, and luciferase activity was evaluated. (**D**) Cell viability was evaluated by flow cytometry at day 4 p.i. in co-infected cells with blood trypomastigotes and controls stained with PI and Annexin-V-FITC. During the analysis 20000 events were acquired and the analysis includes all the ungated cells; percentages of PI and Annexin-V positive cells are indicated. Results are representative of 3 independent experiments performed with cells from different donors.

To evaluate the consequences of co-infection in the early steps of viral cycle, MDM were infected with single round VSV-G and BaL pseudotyped HIV-1, following the infection schemes described above, and luciferase activity was evaluated. *T. cruzi* trypomastigotes induced a significant reduction of luciferase level for both pseudotypes in all the infection schemes (*p<*0.001), being *T. cruzi*-HIV (>99%) stronger than HIV-*T. cruzi* (∼90% for BaL and ∼85% for VSV-G) infection (**Fig. 3A**). No inhibition was detected when blood from *T. cruzi* uninfected mice was used as control (data not shown). When BaL pseudotyped virus was used and TcSn replaced *T. cruzi* trypomastigotes, no luciferase activity was detected (>99% inhibition) regardless of the scheme used (**Fig. 3B**). However, levels of inhibition for VSV-G in the presence of the TcSn were weaker compared with those obtained from the parasite. Differences were also observed with the scheme used. The highest inhibition was obtained when TcSn was administered 24 h earlier than HIV (∼90%), and the weakest inhibition when HIV was administered 24 h earlier than TcSn (*p<*0.001) (**Fig. 3B**).

Taken together, these results show that *T. cruzi* trypomastigotes as well as soluble factors excreted/secreted by them are able to inhibit HIV-1 on the early steps of the replication cycle, suggesting that this effect may occur both at entry and post-entry steps of HIV-1 in MDM.

In order to discard possible effects of mouse blood products remaining in the blood trypomastigote suspension, luciferase activity experiments were carried out with tissue-culture-derived trypomastigotes, grown in Vero cells and harvested from the second passage. Since similar viral inhibition was obtained with blood and tissue-culture-derived trypomastigotes (>99% for VSV-G and ∼93% for BaL) (**Fig. 3C**), the effect on HIV-1 replication induced by *T. cruzi* was confirmed. To rule out any possible effect on cell viability that might interfere with the evaluation of HIV-1 replication, single-infected and co-infected MDM cell viability was evaluated by flow cytometry, using PI and Annexin-V at day 4, the same time in which luciferase activity was assayed. Percentages of PI and Annexin-V positive cells were 9.5% for control MDM, 11% for viral infected MDM, 15.6% for trypomastigote infected MDM and 10.7% for co-infected MDM (**Fig 3D**)**.** These results demonstrate that viability was not affected by infections or co-infections.

### Inhibition of HIV-1 Replication Does Not Depend on the *T. cruzi* Strain

**Figure 4:**
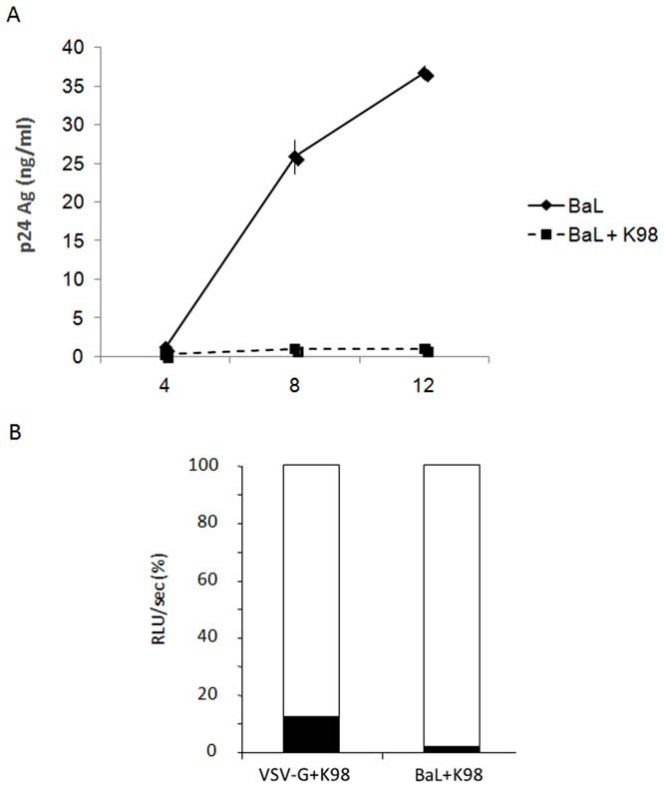
HIV-1 inhibition by a different *T. cruzi* strain. (**A**) MDM were infected simultaneously with HIV-1_BaL_ and K98 clone blood trypomastigotes overnight and p24 antigen production was measured in supernatants. Results are expressed as mean ± SD of triplicates of infection and a representative experiment of 3 others is shown. (**B**) Cells were infected with BaL and VSV-G pseudotyped viruses and K98 strain blood trypomastigotes overnight. At day 4 p.i., cells were lysed and luciferase activity was measured from lysates. Results are expressed as relative light units per second (RLU/sec), presented as a percentage relative to the control (100%), where the histogram in white corresponds to the percentage of infection with the respective control virus and the histogram in black corresponds to the percentage of infection in the presence of the parasite. Results are representative of 4 independent experiments performed with cells from different donors.

Differences in the ability to replicate in macrophages [27] and induction of immune response and genomic characteristics [28] have been reported for *T. cruzi* strains in experimental models. These characteristics might have a differential effect on viral replication. To analyze this hypothesis, MDM were also infected with trypomastigotes of the K98 clone, which has different characteristics from those of the VD strain, and the fully infectious isolate HIV-1_BaL_, simultaneously. P24 antigen production was measured at days 4, 8 and 12 of culture supernatant. **Fig. 4A** shows that p24 production was inhibited in the presence of K98 parasites. MDM were also infected with VSV-G or BaL pseudotypes, and K98 trypomastigotes simultaneously and luciferase activity was evaluated 96 h p.i. Results showed that early steps of the replication cycle were also significantly inhibited by K98 (*p<*0.01) for both pseudotypes with inhibition values of ∼86% for VSV-G and ∼98% for BaL (**Fig. 4B**). These results indicate that *T. cruzi* inhibition of HIV replication does not depend on the parasite strain.

### Early Post-Integration Steps Are Inhibited in Co-Infected MDM

**Figure 5:**
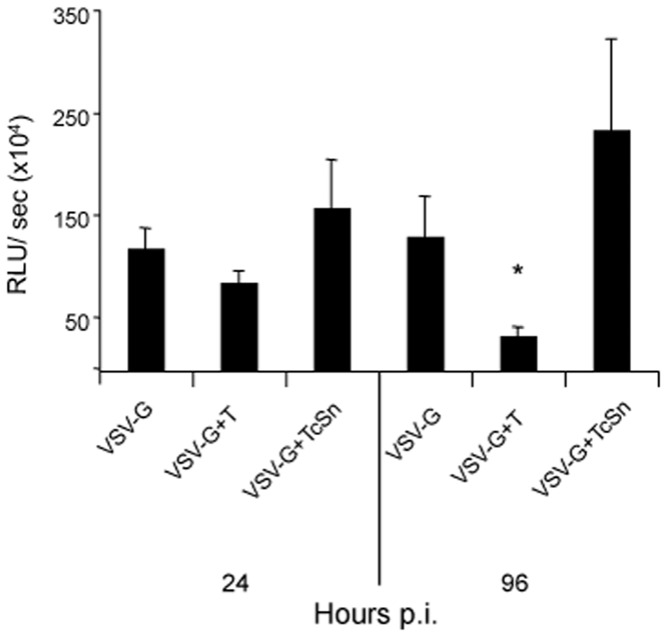
Effect of *T. cruzi* on post-integration events. MDM were infected with VSV-G pseudotyped virus overnight and kept in culture for four days. Then, they were infected with trypomastigotes or treated with TcSn overnight. Cells were lysated 24 or 96 h after parasite infection and luciferase activity was measured in cell lysates. Results are expressed as mean ± SD of RLU/sec of triplicates of infection and a representative experiment of 3 is shown. * *p*<0.01.

The influence of trypomastigotes or TcSn on post-integration steps, including transcription, was analyzed. MDM were infected with VSV-G pseudotype and 96 h later with trypomastigotes or treated with TcSn. Under these conditions, most of the viral DNA should be integrated, which allowed to evaluate the effect of the parasite in post-integration steps [29]. When luciferase activity was measured 24 h and 96 h p.i. or treatment, a reduction of luciferase levels in the presence of the parasite was observed, being significant at 96 h (*p*<0.01), while no differences were found when TcSn was used (**Fig. 5**). These results suggest that trypomastigotes, unlike their excreted/secreted antigens, might affect HIV-1 transcription and protein synthesis.

### Trypomastigotes and TcSn Inhibit Early Post-Entry Steps

**Figure 6:**
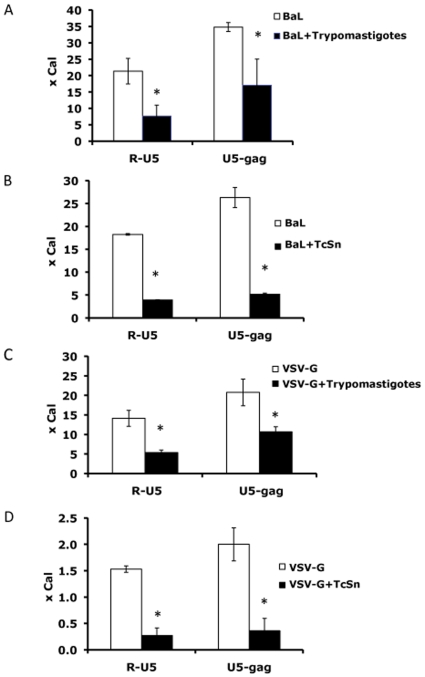
Inhibition of HIV entry and reverse transcription by *T. cruzi*. MDM were infected with BaL pseudotyped virus in the presence of (**A**) trypomastigotes or (**B**) TcSn or VSV-G pseudotyped virus in the presence of (**C**) trypomastigotes or (**D**) TcSn, overnight. Single viral infections with both pseudotypes were performed as control. DNA was isolated and early (R-U5) and late (U5-*gag*) transcripts were quantified by real-time PCR 24 h p.i. Results are expressed as an n-fold difference with respect to the calibrator (x Cal) ± SD of infection duplicates. Results are representative of 3 independent experiments performed with cells from different donors. * *p<*0.05.

As the decrease in early steps of viral replication observed with pseudotyped viruses could be reflecting an effect on steps prior to integration, we evaluated whether reverse transcription and viral entry were affected by *T. cruzi*. Early products of the reverse transcription reflect the input virus entered into the cells [29]. Thus, the influence of the parasite at this level was evaluated by measuring intermediate products of HIV-1 replication through quantitative real-time PCR 24 h p.i. Single round infections with BaL pseudotyped virus and trypomastigotes or TcSn were performed on MDM. The expression of both R-U5 and U5-gag transcripts was significantly diminished in the presence of trypomastigotes or TcSn (*p<*0.05) (**Fig. 6A and 6B**).

Then, in order to elucidate if this decrease represented only viral input or if it was also caused by an impairment of reverse transcription, cells were infected with VSV-G pseudotype virus which by-passes HIV entry process in the presence of live trypomastigotes or TcSn. Levels of both transcripts were diminished for VSV-G, suggesting that reverse transcription would also be affected (**Fig. 6C and 6D**).

These results indicate that entry and early post-entry steps are partially inhibited by the parasite and its soluble factors present in the TcSn.

### *T. cruzi* Modifies Expression of HIV-1 Co-Receptor CCR5 Unlike CD4 Receptor

The inhibitory effect observed on the BaL pseudotyped virus was stronger than that observed on the VSV-G, both in the presence of live trypomastigotes and TcSn. The fact that viral entry is also diminished, it suggests an additional inhibitory effect on the CCR5-dependent viral entry in MDM. To evaluate this hypothesis, cell surface expression of CCR5 and CD4 was measured in cells infected or co-infected overnight with the wild-type BaL isolate and live trypomastigotes or TcSn.

**Figure 7:**
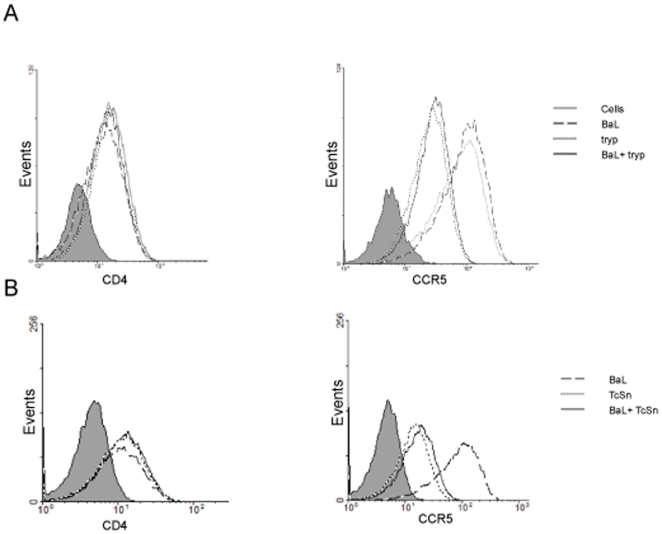
CCR5 expression impaired by *T. cruzi*. Expression of the cell surface of CCR5 and CD4 was evaluated on MDM infected or coinfected with (**A**) *T. cruzi* trypomastigotes or (**B**) TcSn. After overnight infection, cells were harvested, and CCR5 and CD4 expressions were assayed by flow cytometry. Histograms in grey correspond to isotype control. Results are representative of 4 independent experiments performed with cells from different donors.

Surface expression of CCR5 was affected by both trypomastigotes and supernatants, while CD4 expression was not modified. Trypomastigotes, either in the presence of the virus or not, caused a decrease of CCR5 expression of ∼60% and ∼70% respectively (**Fig. 7A**). Similarly, TcSn alone or in combination with the virus caused a reduction of ∼60% for both conditions respectively, when compared with virus-infected cells (*p<*0.05) (**Fig. 7B**). These results indicate that CCR5 expression in cell membrane is inhibited by the presence of trypomastigotes or its soluble factors, suggesting that this would be one of the explanations for HIV-1 replication impairment in MDM.

### Cruzipain, a Major *T. cruzi* Antigen, Is Related to Viral Replication Impairment

**Figure 8:**
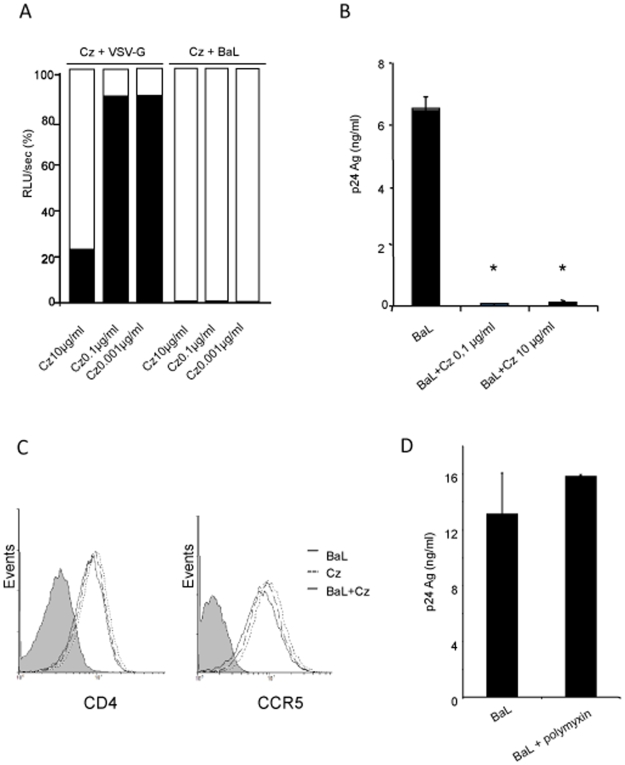
Implication of the major antigen cruzipain in the inhibition of HIV-1 replication cycle. (**A**) Cells were infected with either VSV-G or BaL pseudotyped viruses in the presence of 0.001; 0.1 and 10 µg/ml of cruzipain (Cz). Luciferase activity was measured in cell lysates 96 h p.i. Results are expressed as relative light units per second (RLU/sec), presented as a percentage relative to the control (100%), where the histogram in white corresponds to the percentage of infection with the respective control virus and the histogram in black corresponds to the percentage of infection in the presence of the parasite. A representative experiment of 3 is shown. (**B**) Production of p24 antigen at day 8 p.i. in cells treated with 0.1 and 10 µg/ml of cruzipain. Results are expressed as mean±SD of triplicates of infection and a representative experiment of 3 others is shown. (**C**) Influence of cruzipain on cell surface expression of CD4 and CCR5 after overnight infection in the presence of 0.1 µg/ml of cruzipain. Histograms in grey correspond to isotype control. Results are representative of at least 3 independent experiments. * *p*<0.01. (**D**) Cells were infected with BaL isolate in the presence or absence of polymyxin, and p24 antigen production was measured at day 8 p.i. Results are expressed as mean±SD of triplicates of infection and a representative experiment of 3 is shown.

Cruzipain, the major cystein-protease of *T. cruzi*, is found in every parasite stage but it is only shed to the media by trypomastigotes [30]. In the parasite, cruzipain accumulates in lysosomes near the flagellar pocket, is present at surface level, and shed to the supernatant in parasite cultures. Since parasitic cystein-proteases have been involved in parasite immunoevasion and cell and tissue invasion [31], we analyzed whether cruzipain is involved in the inhibition of the viral replication impairment observed in MDM. The influence of three concentrations (0.001; 0.1 and 10 µg/ml) of cruzipain were assayed on transcriptional activity of VSV-G and BaL pseudotyped viruses. Cruzipain inhibited BaL luciferase activity in all concentrations assayed while it only had a significant effect on VSV-G at higher concentrations (*p<*0.01) (**Fig. 8A**). The production of p24 antigen at day 8 p.i. was also evaluated in cells treated with 0.1 and 10 µg/ml of cruzipain. Both concentrations were responsible for over 90% of the inhibition of antigen production (*p<*0.01) (**Fig. 8B**). Conversely, neither CD4 nor CCR5 cell surface expressions were modified by cruzipain (**Fig. 8C**).

Since *E. coli*-produced recombinant cruzipain could contain endotoxin, polymyxin was added to the protein preparation. Also, considering that polymyxin is a PKC inhibitor which might itself cause the effects ascribed to cruzipain, additional controls were included in which viral infection of MDM were performed with polymyxin in the absence of cruzipain. In this case, p24 antigen production was not affected with respect to that observed in HIV infected MDM (**Fig. 8D**) suggesting that inhibition is mediated by cruzipain itself and not by polymyxin content, if any.

These results indicate that cruzipain is one of the parasite molecules responsible for the inhibition of HIV-1 replication cycle in human macrophages.

## Discussion

As a result of the rapid spread of the HIV pandemic, a number of epidemiological, biological, and clinical interactions between HIV and other tropical pathogens gained relevance. Each pathogen has the potential to alter the epidemiology, natural history, and/or response to therapy of other pathogens [3]. Therefore, it is unpredictable to establish the outcome of such co-infections. In Latin America, one of the most important endemic protozoonoses is Chagas disease and its association with HIV-1 appears to be a substantial threat in large cities, where the distribution of both pathogens overlaps, mostly as a result of the massive migration of rural populations to big cities [13].

Several clinical studies on co-infected patients have reported *T. cruzi* reactivation and manifestation of Chagas disease mainly in AIDS patients [13]. However, few data are available on HIV infection outcome in co-infected subjects; only one case report described the concomitant aggravation of parasite and viral infection in an HIV-positive patient [32].

Much less is known about the interaction of these pathogens at cellular level. We recently reported that *T. cruzi* inhibited HIV replication in human chorionic villi histocultures and in a trophoblast cell line. The study suggested that coinfection with *T. cruzi* may have a deleterious effect on HIV-1 transduction and thus, it could play an important role in viral outcome at the placental level [26]. Here, we have conducted studies of HIV/*T. cruzi* co-infection in their common cellular target: human macrophages. Indeed, macrophages are competent host cells and play critical roles in both pathogens. In *T. cruzi* infection, non-activated macrophages are host cells and contribute to parasite dissemination, while activated macrophages are involved in innate response to infection [33]. In HIV infection, macrophages are among the first cells to be infected since viral infection is spread throughout the body [34], and may therefore persist in tissues for long periods of time and contribute to the propagation of the viral infection [4]-[6].

An important finding of this study is that *T. cruzi* inhibits the HIV-1 replication cycle in lymphoid cells and macrophages. In the model of early infection set in MDM, we found that live trypomastigotes from two different strains of live trypomastigotes, as well as TcSn harboring soluble factors shed by the parasite, were able to impair viral production (**Fig. 2A, 2B** **and** **4A**). Thus, the inhibitory effect induced by *T. cruzi* does not depend on the parasite strain, and the active cell infection of the parasite is not necessary for viral replication impairment. Moreover, early steps of viral replication were also impaired by both trypomastigotes, whether purified from mouse blood or culture derived (**Fig. 3A, 3C** **and** **4B**), and supernatants (**Fig. 3B**), regardless of the infection scheme for both BaL and VSV-G pseudotyped viruses.

The inhibitory effect of soluble products seemed to be less sustainable than that observed in live trypomastigotes. Our results suggest that while live trypomastigotes affected pre- and post-integration events, soluble parasite products only had an inhibitory effect at pre-integration events (**Fig. 3A, 3B** **and** **5**). However, it is important to highlight that *T. cruzi* supernatants were only added to the culture at the moment of infection while trypomastigotes are continuously in contact with cells as they replicate and re-infect other cells.

The strongest effect observed when the parasite precedes viral infection is found in the early steps of viral infection. Therefore, it is likely that the parasite and its products make the cells less susceptible to viral infection. The stronger inhibition for BaL pseudotype, compared to inhibition with VSV-G pseudotype (**Fig. 3A and 3B**), which enters the cell in a co-receptor independent manner [35], suggests an additional effect on the CCR5-dependent entry into the cell. Indeed, the cell surface expression level of CCR5 co-receptor was affected by both trypomastigotes and TcSn (**Fig. 7**). However, it has been reported that the CCR5 density is not only related to the quantity of virus entering the cell but it is also influential at a post-entry step of HIV-1 replication, including reverse transcription [36]. Moreover, it has been described that HIV-1 internalization in macrophages does not depend on envelope-receptor interactions only [37].

Critical structures inside the cytosol of the host cells are required for viral genome retrotranscription and transfer to the nucleus [38], and they could be altered by the parasite or its soluble factors. Moreover, disruption of the reverse transcription complex by exogenous enzymes or alteration of protein interaction can lead to an impaired HIV-1 replication [39]. Experiments using one-round infections, which prevent overlapping replication cycles, together with quantitative real time PCR, showed that the amount of virus entering the cell and the first products of retrotranscription were affected by the parasite and the supernatants, represented by R-U5 levels 24 h p.i. (**Fig. 6A and B**). Furthermore, levels of late transcripts also decreased for VSV-G pseudotyped virus, confirming that reverse transcription could be also affected by *T. cruzi* (**Fig. 6C and D**). Once a trypomastigote enters the cell, it escapes from parasitophorous vacuole to the cytoplasm and gradually changes into the amastigote form. It has been shown that actin filaments play an important role in parasite adhesion and internalization, as well as during the process of differentiation into its intracellular form [40], [41]. Cytoskeleton disruption after parasite infection has been described in different cell types. However, polymerization of actin filaments is required for cellular retention of the parasite inside the cell [41]. On the other hand, the HIV-1 virion interacts with actin filaments after entering the cell, which contributes to the binding to microtubules and the transport to the nuclear periphery [42]. Thus, modifications in macrophages cytoskeleton due to the presence of the parasite actually might impair viral replication.

Several co-infections have been identified to potencially inhibit HIV replication in different cell types and at different phases of viral replication cycle. Some viral co-infections can modulate HIV-1 entry *in vitro*, mainly by modulating chemokines and chemokine receptors [43]–[45]; and measles virus has been able to interfere with HIV-1 reverse transcription and replication by blocking cell-cycle progression of CD4^+^ T lymphocytes [46]. Furthermore, some studies have identified specific proteins from the co-infected pathogen as responsible for the inhibitory effect on HIV-1 [47]–[49]. *T. cruzi* is known to express and/or secrete effector molecules that control cell entry and intracellular targeting or modulate host cell functions required for survival, multiplication, and dissemination within the host [19]–[22]. Moreover, different *T. cruzi* proteins have been reported in the parasites immunoevasion [50]–[52]. Cystein-proteases are not only key factors to parasite immunoevasion but they are also involved in cell and tissue invasion in many parasitic species [53], and have been described as an efficient candidate antigen for vaccine development [54], [55]. *In vitro*, it has been shown that cruzipain, the major cystein-protease of *T. cruzi*, is capable of modulating the immune response towards a type-2 profile by increasing IL-10 and TGF-β secretion, while it simultaneously decreases IL-12 production [53]. In our model, the polarization of the immune response might be also involved in the inhibition of HIV-1 transcriptional activity and p24 antigen production (**Fig. 8A and B**). Similarly, it has been shown that *Mycobacterium tuberculosis* inhibits HIV-1 replication in MDM at an early post-entry level. This inhibition is mediated by soluble factors, not by CCR5-binding chemokines, but partially by endogenous production of IL-10 [7]. Indeed, IL-10 can inhibit HIV-1 in macrophages at early stages of viral replication without affecting CCR5 expression, and also at post-integration stages. This inhibition is associated with the ability of IL-10 to down-modulate the production of pro-inflammatory cytokines IL-6 and TNF-α [56]. In our study, cruzipain did not affect CCR5 expression (**Fig. 8C**). The effect of cruzipain on viral replication in macrophages is clear but all the analyses have been conducted on an *in vitro* model here. We consider that *in vivo* studies analyzing the direct effect on the viral particle should be conducted in order to evaluate cruzipain as a microbicide. Indeed, the search for microbicides worldwide is very active given their possibilities for primary prevention. However, since TcSn diminished CCR5 expression, in the supernatant of trypomastigotes there could be other parasite products, in addition to cruzipain, affecting the viral replication, which identification needs further research.

The significance of our findings on the evolution of co-infection *in vivo* remains unclear since it is difficult to extrapolate *in vitro* studies to an organism with normal or impaired immune reaction. Chagas disease is characterized by an acute phase of infection yielding high levels of circulating parasites -which sometimes leads to death- followed by an indeterminate phase of infection by *T. cruzi* in which the parasitemia becomes subpatent. A re-activation of chronic Chagas disease usually occurs in HIV-infected individuals and circulating parasites can be detected. Consequently, there are variable levels of parasite/cell interaction during *in vivo T. cruzi* infection, which is also dependent of the infecting strain. A case report described the concomitant aggravation of parasite and viral infection in an HIV-positive patient [32] and other clinical studies of co-infected patients described *T. cruzi* reactivation [57]–[59]. In these cases, *T cruzi* infection was already established in the patients by the time of the virus entry, and parasites were restricted to non-immune cells. *T. cruzi* reactivation occurred years later, with patients presenting immunosuppression due to viral infection. The results presented here are the first reported findings regarding the simultaneous interaction of these pathogens in cells that have the potential to kill and control both infections, and identify cruzipain as one of the components of the parasite that interferes with the HIV-1 replication cycle.

## Materials and Methods

### Ethic Statement

Human PBMCs were isolated from healthy blood donors in accordance with the guidelines of the Independent Ethics Committee, School of Medicine, University of Buenos Aires. A written consent was obtained from all blood donors.

CF1 mice were bred and housed at the animal facilities of the Microbiology Department, School of Medicine, University of Buenos Aires. All procedures requiring animals were performed in agreement with institutional guidelines and were approved by the Independent Ethics Committee, School of Medicine, University of Buenos Aires, and conducted in accordance with the guidelines established by the National Research Council.

### Cell Line

T-lymphoblastoid cell line (SupT1) was used [60]. It was maintained in RPMI 1640 10% FCS (Bioser, Córdoba, Argentina) and 50 U of penicillin/ml, 50 µg of streptomycin/ml (Gibco BRL, USA). This cell line was obtained from the AIDS Research and Reference Reagent Program. This study was approved by the Independent Ethics Committee, School of Medicine, University of Buenos Aires.

### Preparation of Peripheral Blood Mononuclear Cells (PBMCs) and Monocyte-Derived Macrophages (MDM)

Human PBMCs were isolated from healthy blood donors by density-gradient centrifugation on Ficoll-Hypaque (Amersham Pharmacia Biotech, Piscataway, NJ) and maintained in RPMI 1640 10% FCS (Bioser, Córdoba, Argentina) and antibiotics.

Monocytes were purified from PBMCs by adherence to plastic in RPMI 1640 (Gibco BRL, USA) alone. Non-adherent cells were removed after 2 h plating by several washes and were allowed to differentiate into MDM in RPMI 1640 10% FCS (Bioser, Córdoba, Argentina), antibiotics and 10 ng/ml of recombinant GM-CSF (Sigma-Aldrich, St. Louis, MO) for 6 days. After differentiation, MDM were washed, detached with 0.25% trypsin-EDTA (Gibco BRL, USA) and plated in 96 and 24 well plates (Greiner, USA) according to the experiment and maintained without GM-CSF. Cells were stained with anti-CD14 antibody and purity was analyzed by flow cytometry; cultures with >95% of CD14 positive cells were used. All the experiments were performed with cells isolated from at least 3 different donors.

### Viral Strains

For cell-free virus infections of MDM, the R5 tropic primary isolate HIV-1_BaL_ [61] was used. The viral stock was prepared in MDM.

For PBMCs infection, X4-tropic primary isolate HIV-1_Lai_ and X4/R5 primary isolate HIV-1_A204_ [26] were used and for T-lymphoblastoid cell line SupT-1 infections, X4 isolate was used. Virus stocks were prepared in PHA/IL-2-activated PBMCs.

Infectious titers were determined by limiting dilution assay on PHA/IL-2-activated PBMCs and expressed as 50% tissue culture infectious dose (TCID_50_)/ml.

Primary isolates were obtained from the AIDS Research and Reference Reagent Program.

### Viral Pseudotypes

Luciferase reporter viruses were produced as previously described [61] by transiently cotransfecting (Lipofectamine 2000, Invitrogen, USA) 293T cells with the proviral pNL-Luc-E-R+ vector [62], which lacks the *env* gene and has the firefly luciferase gene inserted into the *nef* gene, and the expression vector pCMV harbors the gene coding for either the VSV-G envelope protein or the HIV-1 R5 (BaL) envelope protein. 293T cells were also transfected only with pNL-Luc-E-R+ (Denv pseudotype). Supernatants from 293T cells were harvested 72 h after transfection or cotransfection and p24 levels were measured using a commercial ELISA kit (Murex, UK).

### Trypomastigotes of *T. cruzi* and Parasite Supernatants

Two strains of *T. cruzi* were used: a) VD strain isolated from a case of congenital Chagas disease, lethal for mice, phylogenetic lineage “*T. cruzi* II” [28]; and b) K98 clone [27] derived from and with similar features to the CA-I strain, obtained from a patient with chronic myocardiopathy, non-lethal for mice, phylogenetic lineage “*T. cruzi* I” [28]. Strains were maintained by serial passages in 21-day-old CF1 mice. Bloodstream trypomastigotes were obtained from infected *T. cruzi* mice bled at the peak of parasitemia by cardiac puncture and purified as previously described [27]. In order to discard the possible effect of mouse blood present in the trypomastigote suspension, blood from *T. cruzi* uninfected mice was assayed in parallel in all co-infection experiments.

In order to render tissue culture-derived trypomastigotes, Vero cell monolayers were infected with bloodstream trypomastigotes for 24 h. For co-infection assays, culture-derived trypomastigotes harvested from the second passage were used. All trypomastigotes were gently washed, counted in a Neubauer hematocytometer and resuspended at a concentration of 1×10^7^/ml in RPMI 1640 10% FCS for further use in co-infection assays.

Parasite-free supernatant (TcSn) was obtained from suspensions of 1×10^7^/ml trypomastigotes diluted in RPMI 1640 10% FCS medium and incubated for 24 h at 37°C in 5% CO_2_. To remove parasites and cellular debris, trypomastigote suspensions were pelleted (30 min at 10,000 g) and supernatants were filtered through a 0.22 µm pore-size filter. Filtrated aliquots were stored at −80°C until use. For the experimental assays using TcSn, a volume equivalent to the parasite/cell ratio assayed (5∶1) was used. At least 5 different TcSn preparations were tested.

All assays using parasites or TcSn were performed under sterile conditions and endotoxin levels were analyzed in each preparation. LPS contamination, if any, was lower than the detection limits (<10 units/mg) of the Limulus amoebocyte lysate analysis kit (Whittaker Bioproducts, Walkersville, MD).

### Cruzipain Expression

Recombinant cruzipain was cloned and expressed as previously described [54]. Briefly, cruzipain (residues 122 to 467) was expressed in the *E. coli* BL21-D3 strain host, purified by affinity chromatography using a Ni/NTA Sepharose matrix under denaturating conditions, dialyzed against PBS and stored at −70°C until use. Purity was >95%, as assessed by SDS-PAGE. Endotoxin was removed by using polymyxin B-agarose (Sigma Aldrich, St. Louis, MO). Endotoxin levels in the final purified proteins were <10 units/mg, as determined by using a Limulus amoebocyte lysate analysis kit (Whittaker Bioproducts, Walkersville, MD). Protein concentration was determined by Bradford (Bio-Rad Protein Assay) using bovine serum albumin (Sigma-Aldrich, St. Louis, MO) as standard. Although the protein was depleted of endotoxin, 15 µg/ml of Polymyxin (Sigma Aldrich, St. Louis, MO) was added to cruzipain for *in vitro* studies.

### Co-Infection Assays with Viral Strains

PBMCs activated with PHA/IL-2 were infected with HIV-1_Lai_ (0.01 moi) or HIV-1_A204_ (0.01 moi) and *T. cruzi* trypomastigotes (parasite/cell ratio of 5∶1). SupT-1 cell line was infected with HIV-1_Lai_ (0.01 moi) and *T. cruzi* trypomastigotes (parasite/cell ratio of 5∶1). Cells were infected with both pathogens at the same time and p24 production in the supernatant was evaluated using a commercial ELISA kit (Murex, UK) at day 8 post-infection (p.i.).

MDM were infected with HIV-1_BaL_ (0.01 moi) and *T. cruzi* trypomastigotes (parasite/cell ratio of 5∶1) or TcSn (25 µl). Infections were carried out in three different schemes: HIV 24 h before *T. cruzi* (HIV-*T. cruzi)*, HIV 24 h after *T. cruzi* (*T. cruzi*-HIV), or HIV at the same time as *T. cruzi* (HIV+*T. cruzi*). *T. cruzi* indicates either live trypomastigotes or TcSn. P24 antigen was measured in culture supernatants 4, 8 and 12 days post-viral infection.

Since TcSn contain 10 µg/ml of protein and Cz represent ∼1% of the total protein, for cruzipain experiments, two different concentrations were assayed (0.1 and 10 µg/ml for p24 assay), and cruzipain was added at the same time as virus and p24 antigen production was measured at day 8 post-infection. Infections in presence of 15 µg/ml of polymyxin were also performed as a control and viral production was evaluated.

### Co-Infection Assays with Viral Pseudotypes

MDM (5×10^4^ cells/well) plated in 96-well plates were infected overnight with VSV-G (100 ng p24) or BaL (200 ng p24) pseudotypes and *T. cruzi* trypomastigotes or TcSn. Infection schemes were the same as described above. For cruzipain experiments, three different concentrations of cruzipain were assayed (0.001; 0.1 and 10 µg/ml), and cruzipain was added at the same time as the virus. In all these experiments, cells were cultured for an additional 72 h post-viral infection. Then, cells were washed twice and 50 µl of luciferase lysis buffer (Promega, Madison, WI) per well was added. Luciferase activity was measured in 10 µl of lysate with a luminometer (VERITAS), using a commercially available substrate (Luciferase Reporter Assay System, Promega); data are expressed in relative light units per second (RLU/sec).

### HIV-1 Transcripts Amplification

MDM (35×10^4^ cells/well) plated in 24-well plates were co-infected with both VSV-G or BaL viral pseudotypes and blood trypomastigotes (parasite/cell ratio of 5∶1) or TcSn (25 µl) overnight, and DNA (QIAamp, DNA purification kit, Qiagen, Germany) was isolated at day 1 p.i. Single infections with viral pseudotypes were performed as control. HIV-1 R-U5 and U5-gag fragments were quantified by real-time PCR using SYBR Green Master Mix (Applied Biosystems, USA) on an ABI-PRISM 7500 Sequence Detector (Applied Biosystems). The target DNA sequence was specifically amplified with 300 nM of each primer for R-U5 and U5-gag. R-U5: NEC152 GCCTCAATAAAGCTTGCCTTGA and NEC131 GGCGCCACTGCTAGAGATTT; U5-gag: F592 AGATCCCTCAGACCCTTTTAGTCA and R666 CTTTCGCTTTCAAGTCCCTGTT. DNA levels were normalized by quantifying *actin* gen (endogenous reference). Each sample was run in triplicate. The relative changes in R-U5 and U5-gag expression were calculated using the 2^ΔΔCt^ method [63], This method was used after validation experiments demonstrated that the efficiencies of the target and endogenous reference (*actin*) were approximately equal. Tanget DNA levels were expressed against those in reference 8E5 cells, designed as calibrator, similar for each experiment and run in parallel for each analysis. The calibrator was thus considered to be the 1x sample, and all other quantities were expressed as an n-fold difference with respect to the calibrator (x Cal).

### Flow Cytometry

After differentiation, MDM were plated on low attachment 24-well plates (Corning Costar, NY, USA) and infected or co-infected overnight with wild type virus and trypomastigotes, TcSn or cruzipain (0.1 ug/ml). Then, they were extensively washed and CCR5 and CD4 receptors were quantified by flow cytometry. FITC-, APC- or PE-conjugated mAbs directed to CD14, CD4 and CCR5 were used (BD Pharmingen; San Diego, CA). In all cases, isotype-matched control mAbs were used, and a gate (R1) was defined in the analysis to exclude all nonviable cells and debris, based on forward/scatter dot blot. Analysis was performed using a FACS flow cytometer (FACSCanto, BD) and CellQuest software (BD Biosciences, San Jose, CA). The results are expressed as the mean fluorescence intensity (MFI). For cell viability evaluation, co-infected and control infections were stained with FITC-conjugated annexin-V and Propidium Iodide (PI, BD Pharmingen; San Diego, CA) and analyzed using a FACS flow cytometer (FACSCanto, BD).

### Statistics

All statistical comparisons were performed by using analysis of variance. P-values of <0.05 were considered statistically significant.
